# Supplementary material for: Evolution of median fin patterning and modularity in living and fossil osteichthyans
Source: PLoS One. 2023 Mar 15;18(3):e0272246. doi: 10.1371/journal.pone.0272246 (PMC10016723; doi:10.1371/journal.pone.0272246)
Supplement: S1 Table — For each species, the size series is given with SL or TL (mm) and specimen number. Only D. valenciennesi is not represented by immature specimens. (DOCX) [file pone.0272246.s001.docx]

**S1 Table.**

| **Taxa (unit of length)** | **Size series** |
| --- | --- |
| **Actinopterygii** |  |
| *Elonichthys peltigerus ^1^* (SL) | 17.8 (FMNH PF 7488), 19.8 (FMNH PF 7520), 20.3 (FMNH PF 7497), 20.3 (FMNH PF 7524), 24.5 (FMNH PF 7437), 28.9 (FMNH PF 7493), 29.3 (FMNH PF 7522), 29.4 (FMNH PF 7499), 30.0 (FMNH UF 589), 30.4 (FMNH PF 7487), 31.4 (FMNH PF 8975), 32.3 (FMNH PF 7508), 33.6 (FMNH UF 524), 34.2 (FMNH UF 588), 34.3 (FMNH PF 7502), 34.9 (FMNH PF 9058), 37.0 (FMNH UC 21715), 49.5 (FMNH PF 3783) |
| **Actinistia** |  |
| *Miguashaia bureaui ^2^* (TL) | 64.5 (MHNM 06-1633), 85.0 (ULQ 120), 122.4 (MHNM 06-1318), 188.5 (MHNM 06-41), 400.0 (MHNM 06-494), ? (MHNM 06-1232) |
| *Rhabdoderma exiguum ^1^* (SL) | 30.0 (FMNH PF 12386), 31.9 (YPM 56748), 33.0 (FMNH PF 5521), 33.3 (FMNH PF 7528), 35.0 (FMNH PF 8666), 35.6 (FMNH PF 5760), 37.0 (FMNH PF 5494), 37.1 (FMNH PF 8669), 37.7 (FMNH PF 8673), 38.0 (FMNH PF 9954), 40.8 (FMNH PF 3660), 41.4 (FMNH PF 8663), 41.5 (FMNH PF 12383), 42.6 (FMNH PF 12385), 42.9 (FMNH PF 5660), 44.5 (FMNH PF 9952), 44.6 (FMNH PF 8667), 46.7 (FMNH PF 8674), 48.0 (FMNH PF 7338), 48.8 (FMNH PF 8724), 49.3 (FMNH PF 8675), 51.7 (FMNH PF 7529), 52.2 (FMNH PF 8888) |
| **Porolepiformes** |  |
| *Quebecius quebecensis ^2^* (TL) | 47.5 (MHNM 06-1474); 58.67 (KUVP 10891); 173.4 (CMN 4328); 178.9 (MHNM 06-1244) |
| **Dipnoi** |  |
| *Dipterus valenciennesi ^3^* (TL) | 103.0 (BMNH P22195); 181.5 (BMNH P22187); 190.0 (BMNH P22189); 206.8 (BMNH P17638); 280.0 (BMNH P17640) |
| **“Osteolepiformes”** |  |
| *Eusthenopteron foordi ^2^* (SL, or TL when specified) | 27.4 (MHNM 04-1293P9); 28.3 (MHNM 04-1293P8); 40.8 (MHNM 04-1293P10); 41.6 (AMNH 7687); 43.6 (MHNM 06-528); 44.3 (MHNM 04-1293P13); 44.3 (MHNM 06-535); 49.3 (MHNM 06-1754); 56.0 (MHNM 06-47); 57.9 (AMNH 7650); 69.6 (MHNM 06-1336); 69.6 (MHNM 06-213); 70.3 (MHNM 06-1635); 74.4 (AMNH 5906); 78.3 (MHNM 06-288); 88.9 (MHNM 06-111); 97.9 (MHNM 06-1649); 112.0 (MHNM 06-331); 117.0 (AMNH 5900); 124.6 (MHNM 06-277); 127.2 (MHNM 06-850); 130.3 (MHNM 06-1769); 140.0 (MHNM 06-159); 147.1 (MHNM 06-428); 151.3 (MHNM 06-1526); 151.5 (AMNH 10187); 156.6 (MHNM 06-36); 157.1 (AMNH 20222); 165.5 (AMNH 7673); 176.7 (AMNH 5903); 180.0 (MHNM 06-86); 185.7 (ULQ 574); 187.2 (AMNH 7842); 190.9 (MHNM 06-121); 204.2 (MHNM 06-367); 213.3 (AMNH 20223); 220.6 (MHNM 06-829); 220.6 (AMNH 5895); 227.5 (AMNH 7535); 240.9 (ULQ 729); 255.0 (MHNM 06-53); 295.4 (MHNM 06-128); 430.0 TL (MHNM 06-1809); 555.0 (MHNM 06-1219) |

Institutional abbreviations: AMNH, American Museum of Natural History, New York, NY; BMNH, The Natural History Museum, Palaeontology Division, London, UK; FMNH, Field Museum of Natural History, Chicago, IL; KUVP, University of Kansas Museum of Natural History, Division of Vertebrate Paleontology, Lawrence, KS; MHNM, Musée d’Histoire Naturelle de Miguasha, Miguasha, QC; CMN, Canadian Museum of Nature, Ottawa, ON; ULQ, Université Laval, QC; YPM, Yale Peabody Museum of Natural History, New Haven, CT.

^1^ Upper Carboniferous (Pennsylvanian); Mazon Creek, IL, USA

^2^ Upper Devonian (Frasnian); Miguasha, QC, Canada

^3^ Middle Devonian (Givetian); Achanarras Quarry, Scotland, UK
